# Lactopontin in a Simulated Infant Formula Protein Matrix Promotes Bone Development via the Gut–Bone Axis in Growing Rats

**DOI:** 10.3390/nu18081265

**Published:** 2026-04-16

**Authors:** Yipin Lyu, Jie Zhang, Chi Cheng, Xue Tang, Pantian Huang, Feitong Liu, Ruibiao Hu, Thom Huppertz, Xinyan Wang, Peng Zhou

**Affiliations:** 1 School of Food Science and Technology, Jiangnan University, Wuxi 214122, China; 2 School of Chemical Engineering and Materials, Changzhou Institute of Technology, Changzhou 213000, China; 3 Biostime (Changsha) Nutrition Foods Limited, Changsha 410219, China; 4 Health and Happiness (H&H) China Limited, Guangzhou 510700, China; 5 International Joint Research Laboratory for Dairy Science and Technology, Jiangnan University, Wuxi 214122, China; 6 School of Food and Nutritional Sciences, University College Cork, T12 YN60 Cork, Ireland

**Keywords:** lactopontin, bone health, *Parabacteroides*, bile acid

## Abstract

Background: Lactopontin (L-OPN) is a pivotal bioactive protein present in breast milk that supports bone development, but its efficacy in a formula matrix is unknown. This study aimed to evaluate the effects of L-OPN-fortified formula on bone growth in a growing rat model and to explore the underlying mechanisms. Methods: Weanling rats (*n* = 8/group) received daily gavage for four weeks: (1) CON—deionized water; (2) PRO—750 mg/kg·BW mixed protein; or (3) L-OPN—750 mg/kg·BW of the PRO formula fortified with L-OPN. Results: The results showed that the formula fortified with L-OPN could significantly increase bone volume and trabecular bone number (*p* < 0.05). Furthermore, both femur length and thickness, as well as overall body length, were significantly increased (*p* < 0.001). In addition, the L-OPN-fortified formula specifically increased the relative abundance of *Bacteroides* and *Parabacteroides* in rat feces (*p* < 0.05). Metabolomic analysis revealed that L-OPN supplementation significantly altered bile acid metabolism, notably increasing serum levels of 12-ketolithocholic acid (12-KLCA), which correlated strongly with bone metrics. Conclusions: These preclinical findings provide a basis for future research in infant formula.

## 1. Introduction

Human milk is the gold standard for infant nutrition, providing not only essential macronutrients but also a suite of bioactive components critical for optimal growth and development [1]. Among these, lactopontin (L-OPN), a highly phosphorylated glycoprotein, is particularly abundant in human milk yet markedly deficient in conventional infant formulas [2]. Accumulating evidence underscores L-OPN’s pivotal role in early-life bone health, with studies demonstrating its ability to directly stimulate osteoblast activity and promote skeletal mineralization [3,4]. This positions L-OPN as a key candidate for fortifying next-generation infant formulas aimed at better mimicking the bone-supportive benefits of breastfeeding.

However, the efficacy of L-OPN must be evaluated within its natural context of protein–protein interactions. In bovine and human milk, L-OPN does not act in isolation; it forms stable complexes with other bioactive proteins, most notably lactoferrin (LF), through electrostatic interactions [5]. The binding of L-OPN and LF has been extensively studied. It has been demonstrated that bovine-source L-OPN and LF form a stable complex at a ratio of 1:3 (dissociation constant, Kd = 10^−6^ M) [6]. The well-characterized L-OPN–LF complex exhibits distinct functional properties compared to its individual constituents: it demonstrates greater resistance to gastrointestinal digestion [7,8] and enhanced binding and uptake by intestinal cells, which in turn promotes intestinal cell proliferation and strengthens immune responses [9,10,11]. Critically, this complexation fundamentally alters the protein’s digestibility and bioactivity profile. Therefore, a significant research gap exists regarding whether L-OPN can retain or even leverage its osteogenic potential when incorporated into a simulated infant formula protein matrix, where its interaction landscape differs from that of native milk. Demonstrating its efficacy in such a complex system is essential for its practical application.

Addressing this gap necessitates moving beyond simple phenotypic observation to investigate the underlying mechanisms. The gut–bone axis has emerged as a central pathway through which dietary factors regulate skeletal metabolism. Gut microbiota are master regulators of host physiology, capable of transforming primary bile acids into a diverse array of secondary metabolites that act as signaling molecules [12]. Recent research has established direct links between specific bile acid profiles and the regulation of both bone formation and resorption [13]. Given that L-OPN can interact with the gut lumen and modulate microbial communities, as evidenced by our previous work showing L-OPN-induced enrichment of Parabacteroides [3,4], a known producer of secondary bile acids [14], a plausible and testable mechanism emerges: dietary L-OPN, even within a complex formula matrix, may promote bone development by remodeling the gut microbiota, thereby altering the bile acid metabolic profile towards a more osteogenic state.

Thus, the present study was designed to address these interconnected questions using a well-established growing rat model. We aimed to (1) determine whether supplementation with L-OPN in a simulated infant formula protein matrix can promote bone development, and (2) investigate the role of the gut microbiota–bile acid–bone axis as a key mechanistic pathway underlying this effect. We hypothesized that L-OPN-fortified protein powder would enhance bone growth via microbiota-driven modulation of bile acid metabolism. This study presents a preliminary exploration of the potential application of bovine L-OPN in infant formula.

## 2. Materials and Methods

### 2.1. Materials

L-OPN (Lacprodan OPN-10, protein content 90%, purity > 95%) was provided by Arla Food Ingredients Group (Beijing, China). Milk protein concentrate (MPC) and whey protein isolate (WPI) were purchased from Agropur Dairy (Edmonton, AB, Canada). Paraformaldehyde was purchased from Servicebio Co., Ltd. (Wuhan, China). The rat bone alkaline phosphatase (BALP) and Type I collagen cross-linked carboxyl-terminal peptide (CTX-I) Enzyme-linked immunosorbent assay (ELISA) kit was purchased from Elabscience Biotechnology Co., Ltd. (Wuhan, China). Animal RNA extraction kit, All-in-one RT SuperMix for qPCR and SYBR Mix were purchased from Vazyme Biotechnology Co., Ltd. (Nanjing, China). The PCR primers (specific sequence shown in Table S1) were purchased from Sangon Biotech Co., Ltd. (Shanghai, China); chromatographically pure methanol, acetonitrile and formic acid were purchased from Thermo Scientific (Waltham, MA, USA).

### 2.2. Animal Experimental Design

All animal experiment protocols were reviewed and approved by the Laboratory Animal Management and Animal Welfare Ethics Committee of Jiangnan University, with the ethics approval number JN.NO 20250515S0560720[268]. Based on the experimental results of similar studies, through the calculation of the G*Power 3.1 software: set α = 0.05, β = 0.8, effect size d = 1.6, it was concluded that at least 8 animals are needed in each group. Twenty-four 3-week-old male SPF-grade Sprague-Dawley (SD) rats were purchased from Charles River Laboratory Animal Technology Co., Ltd. (SCXK (Zhejiang) 2021-0006) (Jiaxing, China). All the rats had free access to the same standard diet and drinking water throughout the experimental period. The ambient temperature was between 20 and 26 °C, and the relative humidity was between 40 and 70%. After adapting to the environment, the rats were randomly divided into three groups (*n* = 8 per group): (1) the control group (CON) was administered deionized water by gavage; (2) the protein matrix group (PRO) was administered 750 mg/kg·BW of a mixed-protein solution by gavage daily; (3) the L-OPN-fortified group (L-OPN) was administered 750 mg/kg·BW of the same protein matrix, fortified with L-OPN, by gavage daily.

The mixed-protein solution for both the PRO and L-OPN groups was formulated to simulate the whey protein-to-casein ratio (~60:40) found in mature human milk, a key compositional feature often referenced in infant formula design [15]. This matrix was prepared by mixing milk protein concentrate (MPC) and whey protein isolate (WPI) in an equal mass ratio. It is important to note that this experimental matrix was designed specifically to model the protein fraction of human milk/infant formula and does not replicate the full nutritional profile of a commercial infant formula, including its energy density, carbohydrate and fat content, vitamin/mineral fortification, or specific processing conditions. All the animals had ad libitum access to a standard, nutritionally complete rodent diet (e.g., AIN-93G) to meet their basal nutritional requirements. The L-OPN group received the identical PRO matrix supplemented with purified L-OPN to achieve a final dose of 15 mg/kg·BW.

The body weight of the rats was recorded weekly. The experiment was ended after continuous intragastric administration for 4 weeks. Feces were collected. Blood was drawn from the abdominal aorta under isoflurane anesthesia and the rats were sacrificed. The tissues of the femur, liver, kidney, spleen and intestine were dissected and collected.

### 2.3. Determination of Serum Bone Metabolism Markers

The measurement of BALP and CTX-I in rat serum was performed using ELISA kits (see Section 2.1) and was performed in accordance with the instructions provided by the manufacturer.

### 2.4. Micro-CT

Micro-CT testing on femurs was performed according to the method previously described [15], with slight modifications. The isolated femurs were fixed in 4% paraformaldehyde solution for 24–48 h and subsequently rinsed three times with PBS solution. The specimens were then stored in 75% alcohol solution at 4 °C until future use. The processed femurs were scanned using the Bruker SkyScan 1276 small animal scanning imaging system (Kontich, Belgium) at a resolution of 18 μm, voltage of 65 kV, current of 385 μA, and a 1 mm Al filter. The scanning mode was high-resolution mode and the scanning time was 4 min. Three-dimensional reconstruction was carried out using scanning data, and the microstructure parameters of the bone tissue in the target area were analyzed by system analysis software.

### 2.5. Determination of Apparent Indicators of the Femur

Fresh femurs separated were weighed using an analytical balance, and the length, width and thickness of the femurs were measured with a vernier caliper. After the femurs were dried at 105 °C to a constant weight, the backbone weight of the femurs was determined.

### 2.6. Determination of Mechanical Properties of the Femur

The TA.XT plus texture analyzer (Stable Micro Systems, Godalming, UK) was used for the three-point bending of femurs according to the method previously described [16]. Briefly, a 12 mm span (HDP/3PB fixture) and a cross-head speed of 0.6 mm/min were used, with the load applied consistently to the mid-diaphysis. Load–deflection curves were analyzed to obtain the elastic limit load, peak load (N), and the deflection at peak load (mm).

### 2.7. RNA Extraction from Bone Tissue and qPCR

The total RNA of the bone tissue was extracted using the FastPure Plant Total RNA Isolation Kit, with the operation carried out in accordance with the manufacturer’s instructions. The purity and concentration of the extracted RNA were determined using Nanodrop. Complementary DNA was synthesized from qualified samples using HiScript III All-in-one RT SuperMix (Vazyme Biotech, Nanjing, China). DNA was obtained after 20 μL of the reaction system was subjected to a reaction procedure at 50 °C for 15 min and 85 °C for 5 s. A RT-qPCR analysis was performed using SYBR Mix (Vazyme Biotech, Nanjing, China). Pre-denaturation was carried out at 95 °C for 5 min, denaturation for 15 s, and annealing for 30 s at 60 °C. The number of cycles was 40. Taking β-actin as the internal reference gene, relative quantification was performed using the 2^−ΔΔCt^ method [17]. The primers used in the experiments of this chapter are shown in Table S1.

### 2.8. Amplification of 16S rRNA V3-V4 Gene, Library Construction and Sequencing Analysis

The experimental procedure was conducted according to the method of previous studies [18,19]. Fecal DNA from the rats was extracted using a commercial kit and assessed for purity and concentration via agarose gel electrophoresis. An aliquot of each DNA sample was diluted with sterile water to 1 ng/μL. A polymerase chain reaction (PCR) was performed using the diluted genomic DNA as template; barcode-indexed specific primers; a High-Fidelity PCR Master Mix with GC Buffer; and a high-efficiency, high-fidelity enzyme. The resulting PCR products were electrophoresed on a 2% agarose gel, and those that passed quality control were purified using magnetic beads. The purified products were quantified enzymatically, and the samples were pooled in equal amounts based on their respective PCR product concentrations. The pooled mixture was re-analyzed by 2% agarose gel electrophoresis, and target bands were excised and recovered using a Qiagen gel extraction kit (Hilden, Germany).

A sequencing library was constructed with the TruSeq^®^ DNA PCR-Free Sample Preparation Kit (San Diego, CA, USA). The final libraries were quantified using Qubit and quantitative PCR (Q-PCR). Upon passing quality inspection, the libraries were sequenced on the NovaSeq6000 platform. Offline sequencing data were demultiplexed according to the barcode and primer sequences, followed by removal of these adapter sequences. A sequence analysis was performed by the Uparse software (Uparse v7.0.1001, http://drive5.com/uparse/, accessed on 15 August 2025). Sequences with ≥97% similarity were assigned to the same OTUs. Representative sequence for each OTU was screened for further annotation. Amplicon sequence variants (ASVs) were analyzed by Deblur (1.1.0), which uses error profiles to obtain putative error-free sequences from the Illumina sequencing platform. For each representative sequence, the Silva Database (http://www.arb-silva.de/) was used based on the Mothur algorithm to annotate taxonomic information. In order to study the phylogenetic relationship of different OTUs, and the difference in the dominant species in different samples (groups), multiple sequence alignments were conducted using the MAFFT (v7.490, https://mafft.cbrc.jp/alignment/software/, accessed on 15 August 2025). OTU abundance information was normalized using a standard of sequence number corresponding to the sample with the fewest sequences. Subsequent analyses of alpha diversity and beta diversity were all performed based on this output-normalized data. A sample diversity analysis was conducted with the phyloseq (v1.40.0) and vegan (v2.6.2) packages in R (v4.2.0). LEfSe (v1.1.2) was used for linear discriminant analysis effect size (LEfSe) analysis, with the LDA score threshold set to 2. Intergroup differences in species abundance were assessed using *t*-tests in R, and corresponding plots were generated.

### 2.9. Determination of Metabolomics in Serum

Metabolomics analysis in serum was conducted according to the method described in previous studies [20,21]. After thawing on ice, the serum samples were vortexed for 10 s to ensure homogeneity. A 50 μL aliquot was transferred into a labeled centrifuge tube, followed by the addition of 300 μL of 20% acetonitrile in a methanol solution containing internal standards. The mixture was vortexed for 3 min. Both the fecal and serum samples were then centrifuged at 12,000× *g* for 10 min at 4 °C. A 300 μL portion of the supernatant was transferred to a new labeled tube and recentrifuged under the same conditions for 3 min. Finally, 200 μL of supernatant was collected into a corresponding vial insert for LC-MS analysis.

All the samples were analyzed using two complementary LC-MS methods. Chromatographic separation was performed on a Waters ACQUITY Premier HSS T3 column (1.8 μm, 2.1 × 100 mm) with mobile phase A (0.1% formic acid in water) and B (0.1% formic acid in acetonitrile). The MS section alternated between full-scan MS and data-dependent MS^n^ scans using dynamic exclusion. Analysis was conducted on a Q Exactive HF-X mass spectrometer equipped with electrospray ionization in both positive and negative modes. Full scan spectra were acquired from *m*/*z* 75–1000 at a resolution of 35,000.

Raw data were converted to the mzXML format using ProteoWizard. Peak detection, alignment, and retention time correction were performed with XCMS. Peaks with a missing rate > 50% within any sample group were filtered out. Missing values were imputed using a KNN algorithm for values with ≤50% missingness, or replaced with 1/5 of the minimum value for those exceeding 50%. Peak areas were normalized by support vector regression (SVR).

An unsupervised PCA (principal component analysis) was performed by the statistics function prcomp within R (www.r-project.org). The data was unit variance-scaled before the unsupervised PCA. For two-group analysis, differential metabolites were determined by VIP (VIP > 1) and *p*-value (*p*-value < 0.05, Student’s *t* test). VIP values were extracted from the OPLS-DA result, which also contains score plots and permutation plots, and was generated using the R package MetaboAnalystR. The data was log-transformed (log2) and mean-centered before OPLS-DA. In order to avoid overfitting, a permutation test (200 permutations) was performed. Identified metabolites were annotated using the KEGG Compound database (http://www.kegg.jp/kegg/compound/, accessed on 15 August 2025), and annotated metabolites were then mapped to the KEGG Pathway database (http://www.kegg.jp/kegg/pathway.html, accessed on 15 August 2025). Significantly enriched pathways were identified with a hypergeometric test’s *p*-value for a given list of metabolites.

### 2.10. Molecular Docking

The molecular structures of the FXR (PDB ID: 3DCT) and TGR5 (PDB ID: 7DW0) were downloaded from the RCSB protein database (https://www.rcsb.org/). Before docking, water molecules and irrelevant atoms were removed. 12-KLCA structures were downloaded from https://hmdb.ca/metabolites, accessed on 25 December 2025. Virtual screening was performed using AutoDock Vina 1.2 [22]. For each protein–ligand complex, the search algorithm generated nine distinct binding poses. The docking grid box was centered on the active site. All the generated poses were ranked by their predicted binding affinity (expressed in kcal/mol). The pose with the most negative docking score was selected as the representative conformation for further analysis, including visualization of binding modes and characterization of non-covalent interactions.

### 2.11. Statistical Analysis

Data are expressed in the form of mean ± standard deviation (Mean ± SD). Normality was assessed using the Shapiro–Wilk test, and homogeneity of variances was evaluated by Levene’s test. Since the data met the assumptions of normality and equal variance, the differences among more than two groups were calculated using one-way ANOVA followed by Duncan’s multiple range test (the figures in Section 3.1, Section 3.2, Section 3.3 and Section 3.4). An unpaired two-tailed Student’s *t*-test was used to compare the means between the two groups (the figures in Section 3.5 and Section 3.6). A *p*-value < 0.05 was considered statistically significant (* *p* < 0.05, ** *p* < 0.01, *** *p* < 0.001). All the statistical analyses were performed using GraphPad Prism version 8.0. Data points that were extreme outliers, defined as those deviating from the intra-group mean by more than ±3 standard deviations, were excluded.

## 3. Results and Discussion

### 3.1. The Effect of L-OPN-Fortified Formula on the Growth Performance of Rats

**Figure 1:**
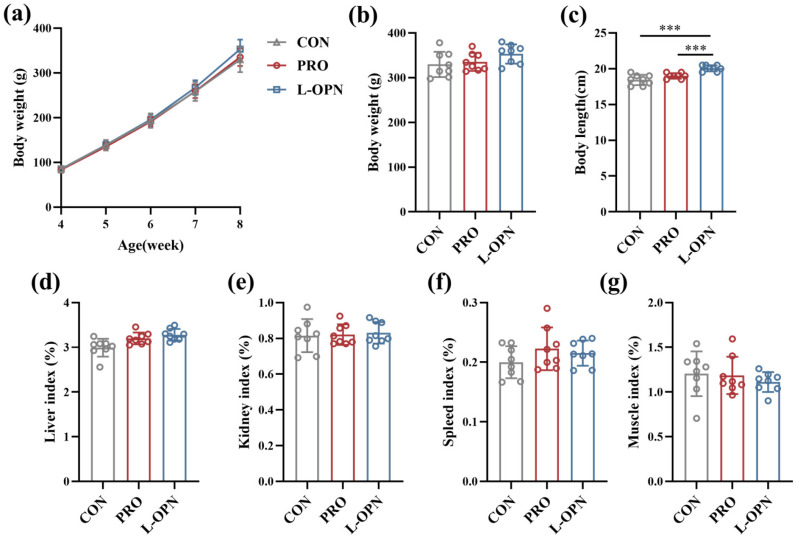
Growth performance of rats in the CON, PRO and L-OPN groups: (**a**) changes in rat body weight; (**b**) he final body weight of rats; (**c**) the body length of rats; (**d**) liver index; (**e**) kidney index; (**f**) Spleen index; (**g**) muscle index; *** *p* < 0.001.

As shown in Figure 1a, the body weight gain trends of the CON, PRO and L-OPN groups were similar throughout the experimental period. The final body weight of the L-OPN group increased by 7.1% and 5.3% compared to the CON and PRO groups, respectively (Figure 1b). However, these differences were not statistically significant (*p* > 0.05). In contrast to body weight, body length for the L-OPN group increased significantly (*p* < 0.001) by 8.8% and 5.6% compared with the CON and PRO groups, respectively (Figure 1c). This selective stimulation of longitudinal growth by L-OPN suggests a targeted anabolic effect on the growth plate rather than a general nutritional boost. Notably, the magnitude of body length increase exceeds what would be expected from protein intake alone, implying that L-OPN may modulate endochondral ossification pathways. This aligns with emerging evidence that L-OPN can directly interact with chondrocytes to enhance proliferation and matrix mineralization during skeletal development [3]. Importantly, the absence of hepatorenal toxicity or immune organ hypertrophy further supports the safety profile of L-OPN fortification in infant formula. The organ index results are presented in Figure 1d–g. No significant differences (*p* > 0.05) were observed among the three groups in terms of the weight ratios of liver, kidney, spleen, or muscle, suggesting that systemic metabolic balance was not affected.

### 3.2. The Effect of L-OPN-Fortified Formula on Serum Bone Metabolism Indicators

**Figure 2:**
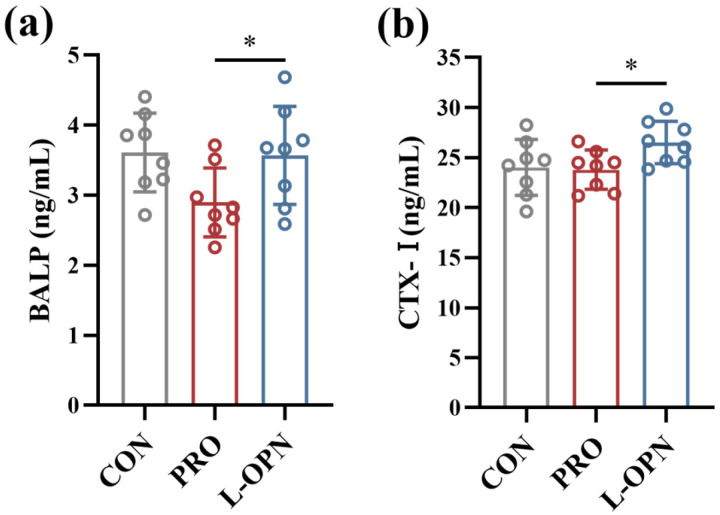
Serum bone metabolism indicators of rats in the CON, PRO and L-OPN groups. The content of (**a**) BALP; (**b**) content of CTX-I; * *p* < 0.05.

Given that body length growth mainly reflects the efficiency of longitudinal bone development, this study evaluated serum bone metabolism markers to further elucidate the underlying mechanism. As shown in Figure 2, serum BALP content in the L-OPN group was significantly (*p* < 0.05) increased by 23.1% compared with the PRO group. In addition, the bone resorption marker CTX-I was also significantly increased, by 11.5% (*p* < 0.05). These results indicate that the L-OPN-fortified formula simultaneously enhances both osteoblastic and osteoclastic activities, thereby comprehensively promoting bone turnover. This pattern aligns with the high-turnover metabolic state characteristic of growing bone and suggests that accelerated bone remodeling provides sustained matrix support for longitudinal bone growth [23].

### 3.3. The Effect of L-OPN-Fortified Formula on Bone Characteristics

**Figure 3:**
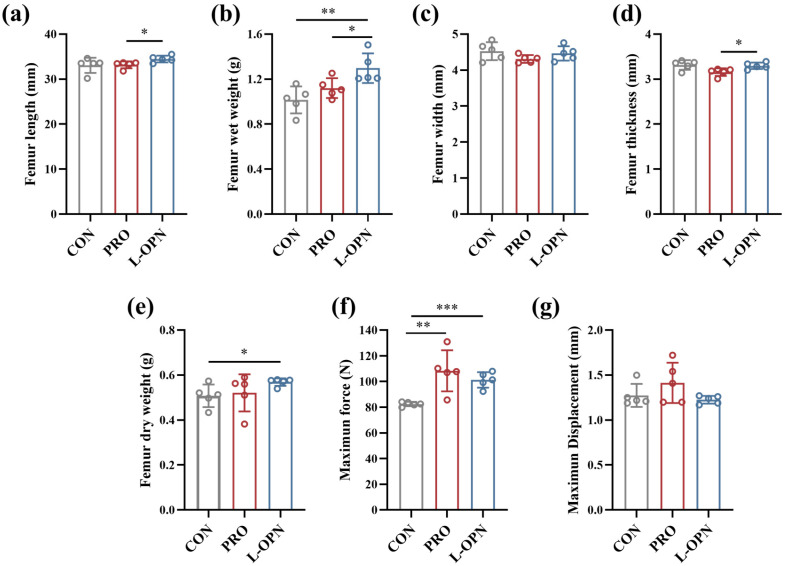
Bone characteristics of rats in the CON, PRO and L-OPN groups. (**a**) Femoral length; (**b**) Femoral wet weight; (**c**) Femoral dry weight; (**d**) Femoral width; (**e**) Femoral thickness; (**f**) Maximum force; (**g**) Maximum displacement; * *p* < 0.05, ** *p* < 0.01, *** *p* < 0.001.

Femoral phenotypic analysis further revealed the unique regulatory mechanism of L-OPN in the mixed protein system. As shown in Figure 3, compared with the CON group, the L-OPN group had significantly (*p* < 0.05) higher femoral mass. Moreover, relative to the PRO group, femoral length, thickness and wet weight in the L-OPN group were significantly higher (*p* < 0.05), by 3.9%, 4.4% and 15.8%, respectively, whereas femoral width and dry weight showed slight, non-significant increases (*p* > 0.05). Of note, L-OPN intervention alone selectively promotes longitudinal growth, increasing length by 5.8% without altering width [4], whereas in the mixed-protein system, both longitudinal and transverse bone development were enhanced simultaneously (Figure 3c,d). This difference suggests that within a breast milk-simulating protein matrix, L-OPN and other milk proteins may release novel bioactive peptides through co-digestion, thereby potentially activating periosteal osteogenesis and contributing to lateral bone mass accumulation.

The biomechanical properties of the femur are critical for motor function and the stability of the skeletal system [24]. As shown in Figure 3f,g, compared with the CON group, the maximum load increased significantly in both the PRO and L-OPN groups (*p* < 0.05). No significant differences were observed in maximum load or maximum deflection between the L-OPN and PRO groups (*p* > 0.05). These results indicate that supplementation with dairy protein enhances bone strength. Moreover, within the protein matrix, L-OPN promoted both longitudinal growth (Figure 3a) and transverse development (Figure 3d) of the femur while maintaining its intrinsic strength (Figure 3f) and toughness (Figure 3g). We hypothesize that the hydrolysis of caseins and/or whey proteins in the presence of L-OPN activates osteoprogenitor cells, thereby stimulating bone growth. This mirrors the bioefficacy of human milk components. The preserved biomechanical properties (Figure 3f,g) confirm that accelerated growth does not compromise structural integrity.

### 3.4. The Effect of L-OPN-Fortified Formula on Bone Microstructure

**Figure 4:**
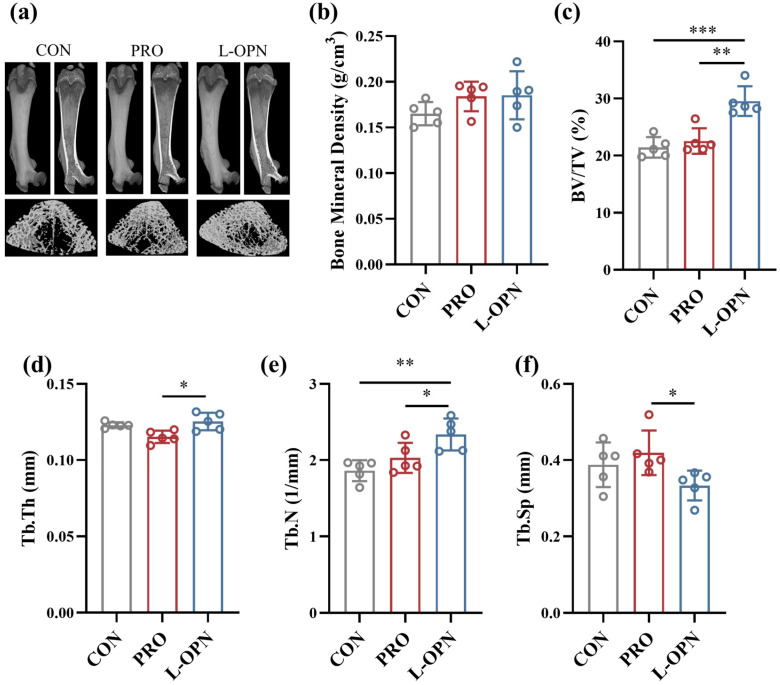
Femoral microstructure of rats in the CON, PRO and L-OPN groups: (**a**) three-dimensional reconstruction image of the femur; (**b**) bone mineral density of rat femur; (**c**) bone volume; (**d**) trabecular bone thickness; (**e**) the number of trabeculae; (**f**) trabecular bone separation degree; * *p* < 0.05, ** *p* < 0.01, *** *p* < 0.001.

Three-dimensional reconstruction and quantitative analysis showed that compared with the CON group, the PRO intervention did not significantly alter femoral microstructure (Figure 4). In contrast, the L-OPN group markedly improved femoral microarchitecture: bone volume fraction increased by 31.0% relative to the PRO group (*p* < 0.01), the thickness and quantity of trabecular bone increased by 8.7% and 15.2% (*p* < 0.05) respectively, and the trabecular separation decreased by 20.5% (*p* < 0.05). Moreover, compared with the CON group, L-OPN significantly increased bone volume (*p* < 0.001) and trabecular bone quantity (*p* < 0.01).

Three-dimensional reconstruction further indicated a pronounced reduction in medullary cavity volume. This pattern exhibits dual regulatory characteristics: similar to L-OPN intervention alone, it stimulates bone volume accumulation by promoting trabecular bone formation; unlike L-OPN alone, it simultaneously enhances the thickness of existing trabeculae. These findings align with previous reports on bovine colostrum protein, containing L-OPN, in mitigating bone loss in ovariectomized models [25]. The observed decrease in medullary cavity volume indicates the occurrence of endocortical apposition, a process that has been demonstrated to enhance the cross-sectional area and bending resistance of the structure [15]. Collectively, these microarchitectural enhancements provide a structural foundation for the observed increases in femoral mass and biomechanical competence, thereby emphasizing L-OPN potential not only in promoting growth but also in preventing age- or disease-related trabecular deterioration.

### 3.5. The Effect of L-OPN-Fortified Formula on Gut Microbiota

**Figure 5:**
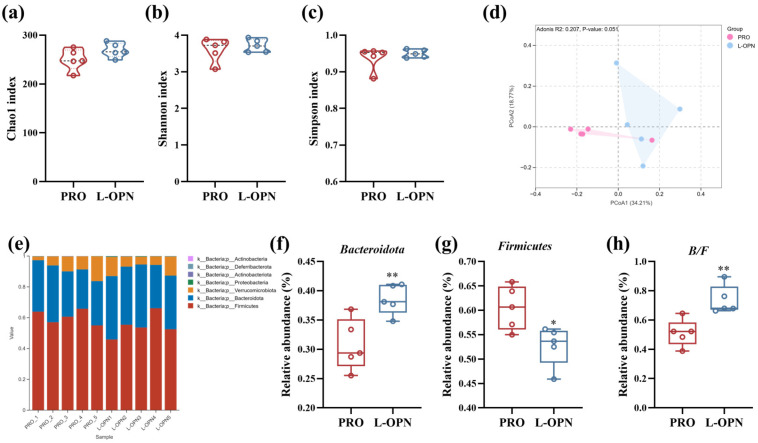
Intestinal flora composition of rats in the CON, PRO and L-OPN groups: (**a**) Chao1 index; (**b**) Shannon index; (**c**) Simpson index; (**d**) β diversity; (**e**) accumulation diagram of microbiota composition; (**f**) relative abundance of *Bacteroidota*; (**g**) relative abundance of *Firmicutes*; (**h**) *Bacteroidota/Firmicutes*; * *p* < 0.05, ** *p* < 0.01.

L-OPN may promote bone growth and development by modulating the intestinal microbiota composition [3]. In this study, we also assessed changes in the gut microbiota of rats following intervention with the L-OPN-fortified formula. The diversity of the composition of intestinal flora in rat feces was analyzed, as shown in Figure 5a–c. α-Diversity is an indicator that describes the richness, uniformity and distribution of species in microbial communities [26], whereas β-diversity quantifies the differences in species diversity among different samples [27]. The L-OPN-fortified formula had no significant effect on the α-diversity indices, i.e., the Chao1, Shannon, and Simpson indices (*p* > 0.05). This was similar to the result that there was no significant change in the α-diversity of the intestinal microbiota in obese pregnant mice after L-OPN intervention [28]. However, L-OPN fortification significantly increased β-diversity (Figure 5d, *p* < 0.05), confirming the overall reconstruction of the microbiota structure. To clarify the specific changes in the intestinal microbiota, the composition at the phylum level was analyzed, with results shown in Figure 5e–h. *Firmicutes* and *Bacteroides* are the two dominant phyla. The abundance ratio (B/F) of these two phyla is often used to assess the balance of the intestinal microbiota. Intervention with the L-OPN-fortified formula significantly increased the abundance of *Bacteroidetes* (by 25.2%, *p* < 0.01), while significantly reducing the abundance of *Firmicutes* (by 12.8%, *p* < 0.05), resulting in a significant increase of 43.6% in the B/F ratio (*p* < 0.01). This indicates that in the complex protein system simulating breast milk, L-OPN-fortified intervention significantly regulates the diversity and composition of the intestinal flora in growing rats.

**Figure 6:**
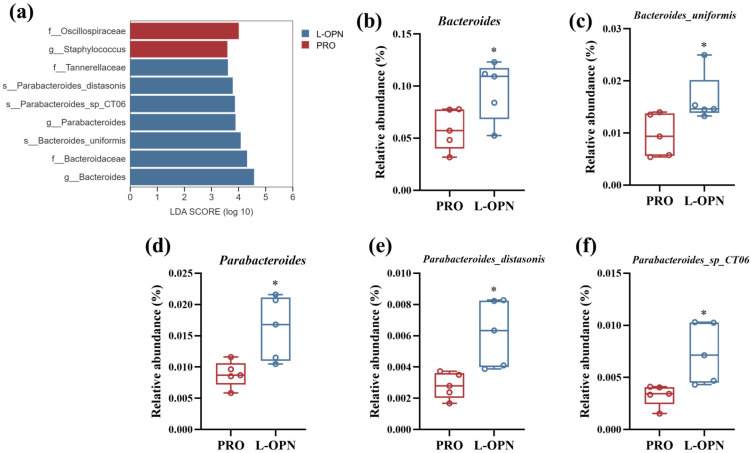
The relative abundance of different bacterial genera/species: (**a**) LEfSe diagram of the microbiota; relative abundance of (**b**) *Bacteroides*; (**c**) *Bacteroides_uniformis*; (**d**) *Parabacteroides*; (**e**) *Parabacteroides_distasonis* relative abundance; (**f**) *Parabacteroides_sp_CT06*. * *p* < 0.05.

The LEfSe analysis further identified the key responding microbiota (Figure 6). In the L-OPN group, the genera that contributed the most to the difference (LDA > 3) were *Bacteroides* and *Parabacteroides*, respectively. The most contributing bacterial species (LDA > 3) were *Bacteroides uniformis*, *Parabacteroides distasonis* and *Parabacteroides sp CT06*. As shown in Figure 6b–f, compared with the control group, the relative abundances of the above-mentioned genera/species in the L-OPN group were significantly increased (*p* < 0.05). Among them, the abundance of *Parabacteroides*, *Parabacteroides_distasonis* and *Parabacteroides_sp_CT06* increased significantly, increasing by 83%, 119% and 123% respectively. This result is consistent with the findings in a previous study that L-OPN intervention promotes an increase in *Parabacteroides* abundance [3], indicating that in the complex protein system, L-OPN can still exert regulatory effects on specific intestinal microbiota.

**Figure 7:**
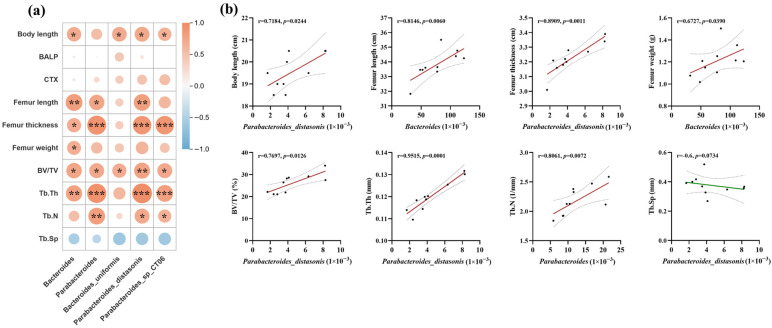
Correlation analysis of differential bacterial genera/species and skeletal indicators: (**a**) Correlation analysis heat map: The color of the graph within the grid reflects the degree of correlation. Red indicates a positive correlation, while blue indicates a negative correlation. The darker the color (the larger the circle), the greater the absolute value of the correlation. Asterisks indicate a significant correlation, with * *p* < 0.05, ** *p* < 0.01, and *** *p* < 0.001. (**b**) Correlation scatter plot, including correlation coefficients and significance, with solid lines representing linear fitting curves and dashed lines representing 95% confidence intervals, every dot represents a sample of a rat.

A correlation analysis was conducted on the important differentially expressed intestinal microbiota and skeletal indicators screened out above. The heat map (Figure 7) showed that, except for serum bone metabolism indicators (BALP, CTX-I) and trabecular bone separation, all the other apparent indicators were significantly positively correlated with the abundance changes in representative intestinal flora genera/species. Among them, *Parabacteroides* showed a very strong positive correlation with bone development indicators: body length (r = 0.72, *p* < 0.05), femoral thickness (r = 0.89, *p* < 0.01), bone volume (r = 0.77, *p* < 0.05), and trabecular bone thickness (r = 0.95, *p* < 0.001). These correlations suggest an association between the genus Parabacteroides (especially *Parabacteroides distasonis*) and bone development. The specific enrichment of Parabacteroides, particularly *Parabacteroides distasonis*, as a known producer of secondary bile acids, is consistent with a potential role between L-OPN, microbial metabolism, and bone anabolism. *Parabacteroides distasonis* has been demonstrated to enhance intestinal barrier function and suppress systemic inflammation [14], both of which are conducive to osteoblast activity [29]. Furthermore, its capacity to deconjugate and transform primary bile acids into bioactive secondary forms [30] may directly influence the bile acid–bone signaling axis identified in our metabolomics data. Our data reveal an associative interaction (L-OPN–*Parabacteroides*–bile acid–bone), which demonstrates the capacity of dietary proteins to manifest systemic effects that are associated with gut microbes.

### 3.6. The Effect of L-OPN-Fortified Formula on Serum Metabolites

**Figure 8:**
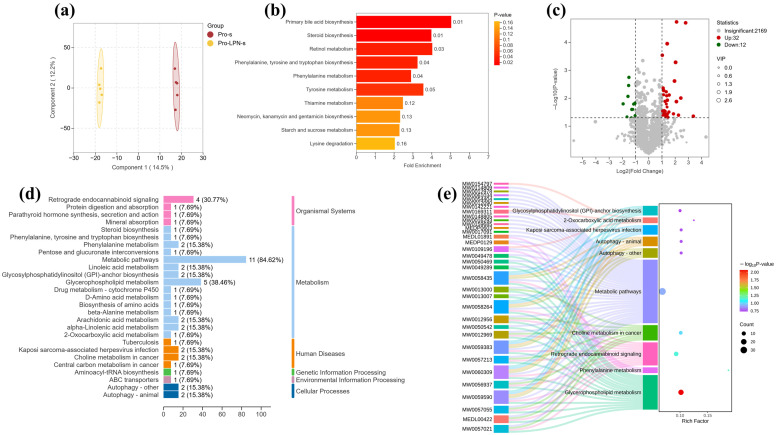
Serum metabolite composition of rats in the PRO and L-OPN groups. Serum metabolite (**a**) OPLS-DA graph; (**b**) MSEA enrichment analysis chart; (**c**) volcano map; classification of serum differential metabolites (**d**) metabolite pathways; (**e**) KEGG Sankey bubble chart.

To explore whether the changes in the composition of the intestinal microbiota after L-OPN intervention would regulate bone metabolism by regulating serum metabolites, non-target metabolomics analysis was conducted on serum samples. The two-dimensional score graph of the orthogonal partial least squares discriminant analysis (OPLS-DA) model shows (Figure 8a) that there are significant differences in the metabolic profiles between the L-OPN-fortified group and the control group. Metabolic set enrichment analysis (MSEA) revealed that the top three metabolic sets in terms of significance were primary bile acid biosynthesis, steroid biosynthesis, and retinol metabolism (Figure 8b). Based on the difference multiple and significance (Fold Change > 2, *p* < 0.05), 32 significantly upregulated metabolites and 12 significantly downregulated metabolites were identified in the L-OPN enhancement group (Figure 8c). Based on the KEGG database annotations, significantly altered metabolites were categorized into functional pathways. As illustrated in Figure 8d, 84.6% of these metabolites are associated with metabolic pathways. The Sankey plot (Figure 8e) also presented the same result, and the significant differences in metabolites were found in the glycerophospholipid metabolic pathway. It was found that only the L-OPN intervention led to an increase in the abundance of *Parabacteroides*, promoting the metabolism of bile acids and resulting in a significant enrichment of serum differential metabolites in the pathways related to fat digestion and absorption (Figure 8c). Therefore, this study further focused on the changes in bile acid metabolism to explore its potential regulatory mechanism on bone metabolism under L-OPN intervention.

**Figure 9:**
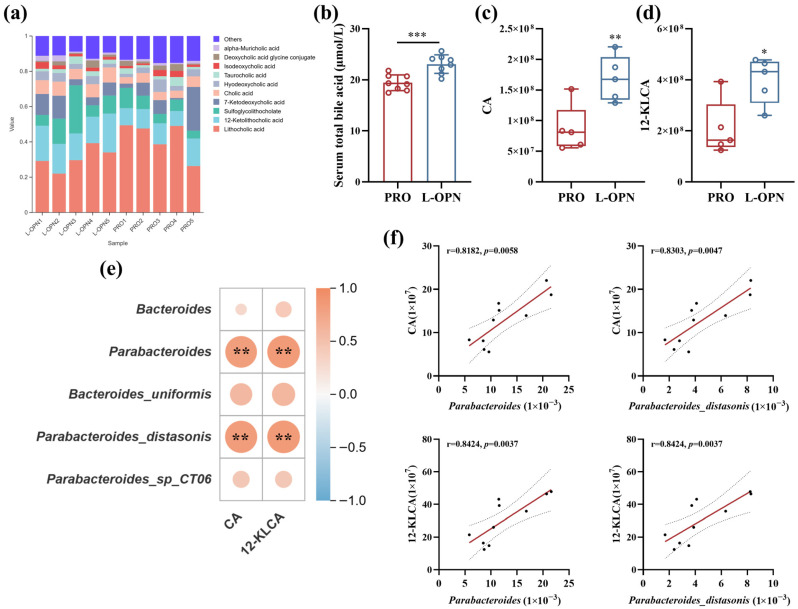
Analysis of the composition of serum bile acids and their correlation with intestinal flora: (**a**) serum bile acid profile; (**b**) serum total bile acid content; (**c**) serum bile acid response value; (**d**) serum 12-ketocholic acid response value; correlation between serum differential bile acids and differential intestinal flora (**e**) heat map and (**f**) scatter plot. The color of the graph within the grid reflects the degree of correlation. Red indicates a positive correlation, while blue indicates a negative correlation. The darker the color, the greater the absolute value of the correlation. Solid lines represent linear fitting curves, and dashed lines represent 95% confidence intervals, every dot represents a sample of a rat. * *p* < 0.05, ** *p* < 0.01, *** *p* < 0.001.

As shown in Figure 9a, a total of 31 bile acids were identified. Among them, Lithocholic acid (LCA) and 12-Ketolithocholic acid (12-KLCA), Sulfoglycolithocholate (SLCG-SO_3_), 7-Ketolithocholic acid (7-KLCA) and Cholic acid (CA) exhibited the highest peak areas, indicating their relatively abundant accumulation or strong ionization response under the analytical conditions. Compared with the CON group, the total bile acid content in the serum of rats in the L-OPN group (23.06 ± 1.85 μmol/L) was significantly increased by 18.7% (*p* < 0.001). The level of CA increased extremely significantly by 94.9% (*p* < 0.01), and the level of 12-KLCA was also significantly upregulated, by 91.1% (*p* < 0.05). CA has physiological activities such as maintaining bile acid homeostasis, improving inflammation, and protecting nerves. Clinically, CA is used to treat cholestatic diseases, which can improve lipid metabolism and prevent liver damage [31]. CA also suppresses osteoclast differentiation by inhibiting the AMPK signaling pathway [32]. 12-KLCA is an anti-inflammatory secondary bile acid derived from the microbiota that targets and regulates group 3 innate lymphoid cells (ILC3s), and it is a potential intervention molecule for ulcerative colitis [33] and metabolic diseases [34]. The gut microbiota are involved in the metabolism of bile acids. The results of the correlation analysis also showed (Figure 9c,d) that the level of bile acids was significantly correlated with the relative abundance of *Bacteroides* (r = 0.82, *p* < 0.01), and was also significantly positively correlated with the abundance of *Parabacteroides distasonis* (r = 0.83).

**Figure 10:**
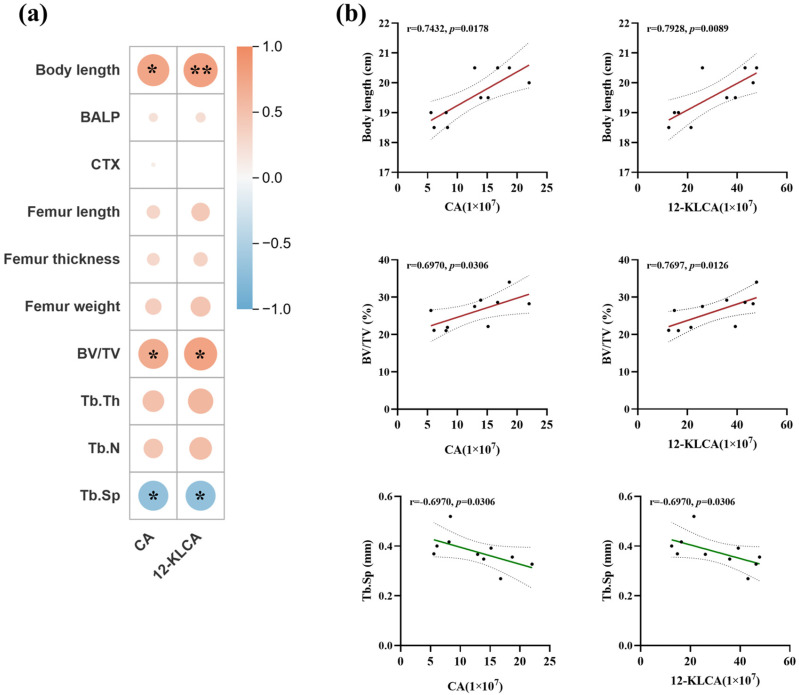
Correlation analysis of differential bile acids and bone indicators. Correlation between serum differential bile acids and femoral indicators (**a**) heat map and (**b**) scatter plot. The color of the graph within the grid reflects the degree of correlation. Red indicates a positive correlation, while blue indicates a negative correlation. The darker the color, the greater the absolute value of the correlation. Solid lines represent linear fitting curves, and dashed lines represent 95% confidence intervals, every dot represents a sample of a rat. * *p* < 0.05, ** *p* < 0.01.

Certain specific bile acids have also been confirmed to have the function of regulating bone metabolism. For instance, chenodeoxycholic acid can activate the differentiation of bone marrow stem cells into osteoblasts mediated by *Runx2* while inhibiting the generation of adipocyte-like phenotypes [35]. Intervention with 200 mg/kg taurodeoxycholic acid for four weeks can significantly increase the femoral bone volume and bone mineral density of mice [36]. Therefore, a correlation analysis was conducted between bile acid levels and bone indicators (Figure 10). The results indicated that body length, bone volume, and trabecular bone separation were significantly correlated with bile acid levels. Among them, the level of 12-KLCA was positively correlated with body length (r = 0.78, *p* < 0.01) and bone volume (r = 0.77, *p* < 0.05), and significantly negatively correlated with trabecular bone separation degree (r = 0.77, *p* < 0.01). This suggests that the observed changes in bile acids, particularly 12-KLCA, may be associated with the metabolic conditions of bone.

The metabolic status of the bile acids was determined, and it was found that the bile acid transport receptors at the terminal ileum also changed (Figure S1). Among them, the gene expressions of *Asbt* and *Ostα* were upregulated by 1.42 times and 0.98 times respectively compared with the control group (*p* < 0.001), indicating that L-OPN promoted the reabsorption of the bile acids and the enterohepatic circulation, and optimized the metabolism of the bile acids. Bone cell membrane receptor *Tgr5* and nuclear receptor *Fxr* are important targets for bile acid regulation of bone metabolism [32].

The results of the molecular docking (Figure S1a,b) showed that 12-KLCA could interact with human TGR5 [37] and FXR [38], with binding energies of −6.1 kcal/mol and −7.8 kcal/mol, respectively. L-OPN enhancement significantly upregulated the expression of bile acid receptor *Fxr* (increased by 1.47 times, *p* < 0.05) and *Tgr5* (increased by 3.59 times, *p* < 0.001) in the femur. It also activated the expression of the downstream transcription factor *Runx2* (increasing by 2.57 times, *p* < 0.001). Ileal bile acid transporters (ASBT and OSTα) and bone FXR/TGR5 receptors create a coordinated signaling loop. 12-KLCA is a microbiota-derived secondary bile acid enriched in our study that correlates strongly with bone metrics and demonstrates favorable binding to TGR5 and FXR. TGR5 activates osteoblast differentiation via cAMP/PKA, while FXR suppresses osteoclastogenesis. Together, these findings position bile acids not merely as metabolic intermediates but as potential endocrine mediators, thereby providing a plausible framework through which gut microbial activity could be translated into skeletal anabolism.

In addition to the gut–bone axis pathway, which was identified in our previous studies [3,4], L-OPN may also be involved in bone regulation by promoting calcium absorption and generating osteogenic peptides. Consequently, we further validate the latter two mechanisms. As shown in Figure S2a–c, the L-OPN-fortified formula significantly upregulated (*p* < 0.05) the expression of genes related to calcium transmembrane transport (*Trpv6*, *Cacnald*, *Atp2b1*) and key protein genes of the cell bypass pathway (*Cldn15*, *Occludin*) in the jejunum of rats. However, no significant change was observed in the blood calcium level (*p* > 0.05), and although the bone mineral content slightly increased, the difference was not significant (*p* > 0.05). The above results indicate that promoting calcium absorption is not the main way for L-OPN to exert its bone-promoting effect. In addition, there was no significant difference in the content of L-OPN derived polypeptide WS7 in the serum of the two groups of rats (*p* > 0.05), and there was no significant correlation between the WS7 level and the body length, femoral length, and bone volume of the rats (*p* > 0.05), indicating that the bone growth promoting effect of the L-OPN-fortified formula was not related to WS7. Despite this, it cannot be ruled out that L-OPN may generate other polypeptides with osteogenic activity in the protein matrix. This hypothesis requires further experimental verification.

## 4. Conclusions

In conclusion, this study provides preliminary evidence that L-OPN-fortified formula is associated with the concurrent modulation of the gut microbiome, bile acid metabolism (notably 12-KLCA), and bone tissue, supporting healthy skeletal development in a juvenile rodent model. However, this study has several limitations that warrant consideration. First, the experiments were conducted in a rodent model; although growing rats are a well-established surrogate for infant skeletal development, translatability to human infants requires validation in clinical studies. Second, while we identified 12-KLCA as a potential mediator, causal evidence linking this bile acid metabolite to osteogenesis was not established; future studies using metabolite supplementation or receptor knockout models are needed. Finally, the protein matrix used herein simulates only key aspects of infant formula and does not fully recapitulate its complex composition (e.g., lipids, carbohydrates, and micronutrients), which may modulate L-OPN bioactivity in real-world formulations. Therefore, while L-OPN presents as a promising multifunctional nutritional component, its functional significance and true potential for infant health remain uncertain and warrant further investigation under more physiologically and clinically relevant conditions.

## Data Availability

The data presented in this study are available on request from the corresponding author due to being a part of an ongoing study.

## Supplementary Materials

The following supporting information can be downloaded at https://www.mdpi.com/article/10.3390/nu18081265/s1: Table S1: Primer sequence; Figure S1: Changes in bile acid metabolism. Molecular docking results of 12-KLCA with (a) TGR5 and (b) FXR; (c) Expression levels of genes related to ileal bile acid transport; (d) Expression levels of femoral bile acid receptors and downstream genes; * *p* < 0.05, *** *p* < 0.001; Figure S2: Changes in calcium and WS7 metabolism in rats. (a) Serum calcium content; (b) Bone mineral content; (c) Expression level of jejunal calcium transporter genes; (d) Serum WS7 content; (d–g) Correlation between the content of WS7 in rat serum and body length, femoral length and bone volume; * *p* < 0.05, ** *p* < 0.01, *** *p* < 0.001.
