# Supplementary material for: Lactopontin in a Simulated Infant Formula Protein Matrix Promotes Bone Development via the Gut–Bone Axis in Growing Rats
Source: Nutrients. 2026 Apr 16;18(8):1265. doi: 10.3390/nu18081265 (PMC13118673; doi:10.3390/nu18081265)
Supplement: Supplementary file 1 [file nutrients-18-01265-s001.zip › nutrients-4214543-supplementary.pdf]

Table S1 Primer sequence

| Gene | Forward primer（5’-3’） | Reverse primer（5’-3’） |
|---|---|---|
| *Bmpr* | AACCTCGTTCAGTAAGGCCG | CGGCCACCTTGATTACTGGT |
| *Smad1* | TGACTGGGAACGGATCGGA | GGTCTTCGGTTCGGAAAGGT |
| *Smad5* | TGTTGGGCTGGAAACAAGGT | GTGACACACTTGCTTGGCTG |
| *Smad8* | CTACCCGCACAACCGGAG | GGTCAGCGGCAAGTATCTGT |
| *Fxr* | CACTGACACGCCCTTTTTGC | TGGAGGATAAAACGAGGCGG |
| *Tgr5* | CCACCACTAGGGCCTGTAAC | CCTCGAAGCACTTGTAGCCA |
| *Runx2* | CACAAGTGCGGTGCAAACTT | AATGACTCGGTTGGTCTCGG |
| *Opn* | CCAGCCAAGGACCAACTACA | AGTGTTTGCTGTAATGCGCC |
| *β-actin* | CCCGCGAGTACAACCTTCTT | CGCAGCGATATCGTCATCCA |

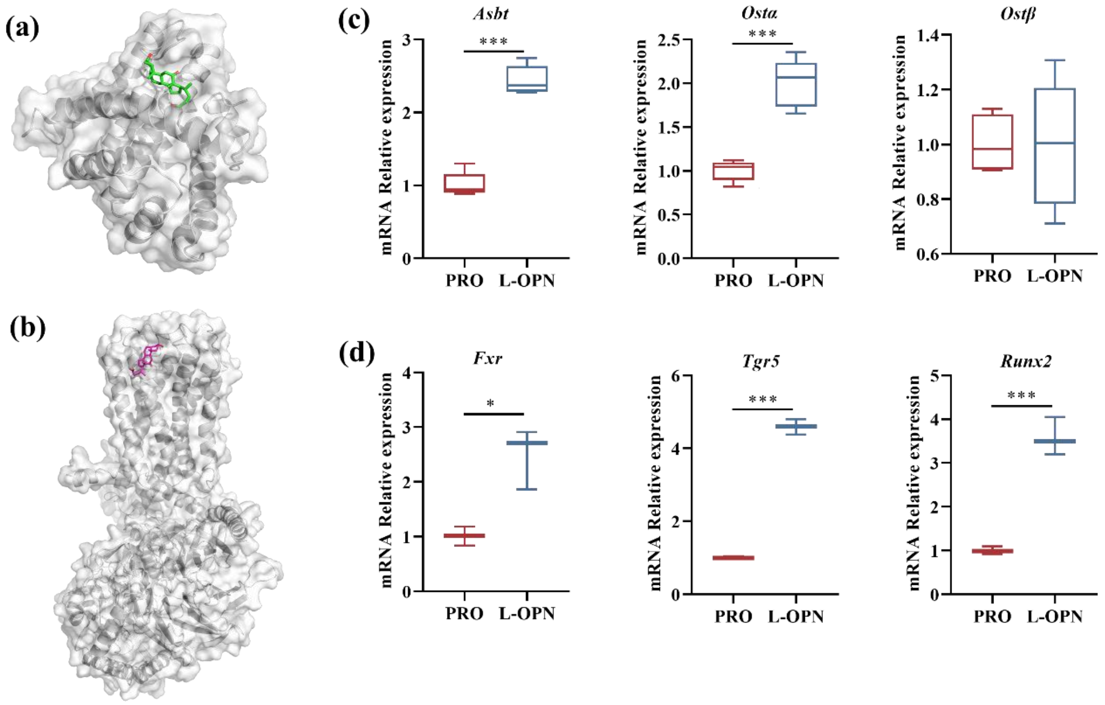

(a)
(b)
(c)
Asbt
Ostα
Ostβ
***
mRNA Relative expression
PRO
L-OPN
(d)
Fxr
Tgr5
Runx2
*


**Figure S1**

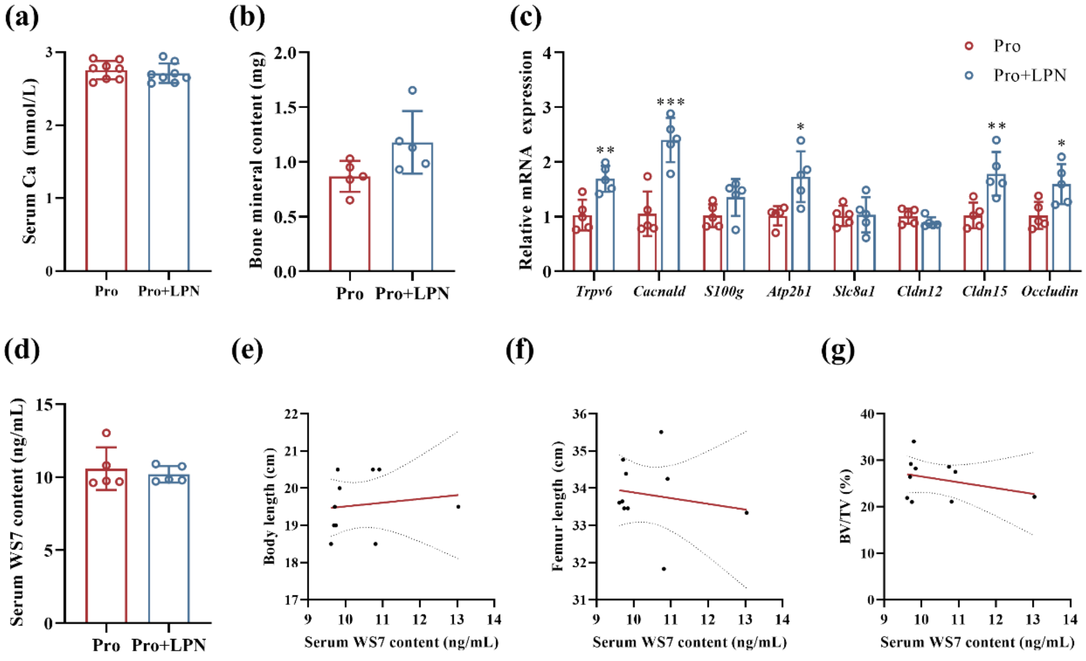
(a)
Serum Ca (mmol/L)
Pro
Pro+LPN
(b)
Bone mineral content (mg)
Pro
Pro+LPN
(c)
Relative mRNA expression
Pro
Pro+LPN
Trpv6
Cacnald
S100g
Atp2b1
Slc8a1
Cldn12
Cldn15
Occludin
(d)
Serum WS7 content (ng/mL)
Pro
Pro+LPN
(e)
Body length (cm)
Serum WS7 content (ng/mL)
(f)
Femur length (cm)
Serum WS7 content (ng/mL)
(g)
BV/TV (%)
Serum WS7 content (ng/mL)

**Figure S2**
